# Supplementary material for: Evolution of tooth morphological complexity and its association with the position of tooth eruption in the jaw in non-mammalian synapsids
Source: PeerJ. 2024 Aug 12;12:e17784. doi: 10.7717/peerj.17784 (PMC11326432; doi:10.7717/peerj.17784)
Supplement: Supplemental Information 1 [file peerj-12-17784-s001.pdf]

Supplementary Information for:

Evolution of tooth morphological complexity and its association with the position of tooth eruption in the jaw in non-mammalian synapsids

Supplementary Text

Supplementary Results

Reconstructed ancestral states of the positioning of dentition

Ancestral state reconstruction showed an unambiguous increase in tooth complexity in nine nodes, a possible increase in tooth complexity in three nodes, an unambiguous reduction in tooth complexity in two nodes, and a possible reduction in tooth complexity in one node in the phylogeny of non-mammalian synapsids (Fig. 1; further details on the tree can be found in Fig. S1).

The dentition position relative to the anterior end of palatine was more posterior in five of the nine nodes with an unambiguous increase in tooth complexity and one of the three nodes with a possible increase in tooth complexity than what was seen in the immediate ancestor (Fig. 4; further details on the tree can be found in Fig. S2). This position was more posterior in the two nodes with an unambiguous reduction in tooth complexity than what was seen in the immediate ancestor, while it was more anterior in the node with a possible reduction in tooth complexity than in the immediate ancestor (Fig. 4).

The dentition position relative to the posterior end of palatine was more posterior in eight of the nine nodes with an unambiguous increase in tooth complexity and two of the three nodes

with a possible increase in tooth complexity than what was observed in the immediate ancestor (Fig. 5; further details on the tree can be found in Fig. S3). This position was more posterior in the two nodes with an unambiguous reduction in tooth complexity and in the node with a possible reduction in tooth complexity than what was observed in the immediate ancestor (Fig. 5).

The dentition position relative to the posterior end of maxilla was more posterior in all of the nine nodes with an unambiguous increase in tooth complexity and in one of the two nodes with a possible increase in tooth complexity, than that observed in the immediate ancestor (Fig. 6; further details on the tree can be found in Fig. S4). This position was more anterior in one of the two nodes with an unambiguous reduction in tooth complexity and in the node with a possible reduction in tooth complexity than observed in the immediate ancestor (Fig. 6).

The dentition position in the cranium was more posterior in all of the nine nodes with an unambiguous increase in tooth complexity and two of the three nodes with a possible increase in tooth complexity than what was observed in the immediate ancestor (Fig. 7; further details on the tree can be found in Fig. S5). This position was more anterior in one of the two nodes with an unambiguous reduction in tooth complexity than what was observed in the immediate ancestor, while it was more posterior in the node with a possible reduction in tooth complexity than in the immediate ancestor (Fig. 7).
