# Supplementary material for: Evolution of tooth morphological complexity and its association with the position of tooth eruption in the jaw in non-mammalian synapsids
Source: PeerJ. 2024 Aug 12;12:e17784. doi: 10.7717/peerj.17784 (PMC11326432; doi:10.7717/peerj.17784)
Supplement: Supplemental Information 4 [file peerj-12-17784-s004.pdf]

Supplementary Information for:

Evolution of tooth morphological complexity and its association with the position of tooth eruption in the jaw in non-mammalian synapsids

Table S1: Estimates of phylogenetic generalized least squares (PGLS) models used to quantify the positioning of dentition in the upper jaw or cranium for the ancestral state reconstructions in non-mammalian synapsids.

Explanatory variable	Estimate	SE	t	P
(a) Dentition position relative to the anterior end of palatine				
Intercept	1.096	0.243	4.506	< 0.001
Length to ante-palatine	0.902	0.057	15.958	< 0.001
(b) Dentition position relative to the posterior end of palatine				
Intercept	0.001	0.209	0.004	0.997
Length to post-palatine	0.986	0.037	26.747	< 0.001
(c) Dentition position relative to the posterior end of maxilla				
Intercept	-0.085	0.182	-0.467	0.642
Length to post-maxilla	0.994	0.033	29.857	< 0.001
(d) Dentition position in the cranium				
Intercept	-0.851	0.225	-3.780	< 0.001
Cranial length	1.041	0.038	27.284	< 0.001

(a) The dentition position relative to the anterior end of palatine was calculated as residuals from the regression of the length to post-dentition (log) on the length to ante-palatine (log) (see Fig. 2 for definitions of positions and length measurements). An estimated λ of 0.527 was used in the PGLS model. (b) The dentition position relative to the posterior end of palatine was calculated as residuals from the regression of the length to post-dentition (log) on the length to post-palatine (log). An estimated λ of 0.928 was used in the PGLS model. (c) The dentition position relative to the posterior end of maxilla was calculated as residuals from the regression of the length to post-dentition (log) on the length to post-maxilla (log). An estimated λ of 0.865 was used in the PGLS model. (d) The dentition position in the cranium was calculated as residuals from the regression of the length to post-dentition (log) on the cranial length (log). An estimated λ of 0.879 was used in the PGLS model.
