# Supplementary material for: Evolution of tooth morphological complexity and its association with the position of tooth eruption in the jaw in non-mammalian synapsids
Source: PeerJ. 2024 Aug 12;12:e17784. doi: 10.7717/peerj.17784 (PMC11326432; doi:10.7717/peerj.17784)
Supplement: Supplemental Information 5 [file peerj-12-17784-s005.pdf]

Supplementary Information for:

Evolution of tooth morphological complexity and its association with the position of tooth eruption in the jaw in non-mammalian synapsids

Table S2: Reconstructed ancestral states of the dentition position relative to the anterior end of palatine at each node, which is numbered in Fig. S2.

Node	State at ancestor	State at node
3	0.03812466	-0.02094084
4	-0.02094084	-0.03497724
5	-0.03497724	-0.12802991
6	-0.12802991	-0.14263149
7	-0.14263149	-0.16819690
8	-0.16819690	-0.16916452
9	-0.16916452	-0.15299115
10	-0.15299115	-0.33348201
11	-0.33348201	-0.35295726
12	-0.35295726	-0.35233014
13	-0.35233014	-0.34588860
14	-0.34588860	-0.09116516
15	-0.09116516	-0.26392405
16	-0.26392405	-0.35400154
17	-0.35400154	-0.37824450
18	-0.37824450	-0.39472631
19	-0.39472631	-0.49581935
20	-0.49581935	-0.50918401
21	-0.50918401	-0.60738440
22	-0.60738440	-0.66058700
23	-0.60738440	-0.34825000
24	-0.50918401	-0.51196200

25	-0.49581935	-0.46833000
26	-0.39472631	-0.45772700
27	-0.37824450	-0.42753868
28	-0.42753868	-0.36671698
29	-0.36671698	-0.31606003
30	-0.31606003	-0.32648730
31	-0.32648730	-0.55887400
32	-0.32648730	-0.09536900
33	-0.31606003	-0.18695700
34	-0.36671698	-0.36628300
35	-0.42753868	-0.48890500
36	-0.35400154	-0.27396200
37	-0.26392405	-0.44339000
38	-0.09116516	0.09770900
39	-0.34588860	-0.41968600
40	-0.35233014	-0.35298795
41	-0.35298795	-0.36592019
42	-0.36592019	-0.40704761
43	-0.40704761	-0.34127765
44	-0.34127765	-0.14016700
45	-0.34127765	-0.16831400
46	-0.34127765	-0.53326300
47	-0.40704761	-0.53120754
48	-0.53120754	-0.13185000
49	-0.53120754	-0.58587400
50	-0.36592019	-0.38375600
51	-0.35298795	-0.25928100
52	-0.35295726	-0.44513500
53	-0.33348201	-0.35534242
54	-0.35534242	-0.34645400
55	-0.35534242	-0.37652300
56	-0.15299115	-0.09015526

57	-0.09015526	-0.22101617
58	-0.22101617	-0.25996545
59	-0.25996545	-0.24501296
60	-0.24501296	-0.20226840
61	-0.20226840	0.26433000
62	-0.20226840	-0.59832400
63	-0.24501296	-0.24543700
64	-0.25996545	-0.54962900
65	-0.22101617	-0.28656843
66	-0.28656843	-1.13452600
67	-0.28656843	-0.21087800
68	-0.22101617	-0.25276429
69	-0.25276429	-0.40973300
70	-0.25276429	-0.14985900
71	-0.09015526	0.36585500
72	-0.16916452	-0.30244110
73	-0.30244110	-0.31414202
74	-0.31414202	-0.30666874
75	-0.30666874	-0.28429723
76	-0.28429723	-0.28013922
77	-0.28013922	-0.27929595
78	-0.27929595	-0.30057011
79	-0.30057011	-0.33981588
80	-0.33981588	-0.41930691
81	-0.41930691	-0.28728300
82	-0.41930691	-0.64303500
83	-0.33981588	-0.27574500
84	-0.30057011	-0.22997900
85	-0.27929595	-0.19753795
86	-0.19753795	-0.17177500
87	-0.19753795	-0.21590100
88	-0.28013922	-0.24984300

89	-0.28429723	-0.17057800
90	-0.30666874	-0.39521100
91	-0.30666874	-0.31211300
92	-0.31414202	-0.43810100
93	-0.30244110	-0.39944366
94	-0.39944366	-0.41402100
95	-0.39944366	-0.40979500
96	-0.16819690	-0.21920144
97	-0.21920144	-0.42959660
98	-0.42959660	-0.79302400
99	-0.42959660	-0.69029400
100	-0.21920144	-0.09034600
101	-0.14263149	0.04531218
102	0.04531218	0.07053621
103	0.07053621	0.05714997
104	0.05714997	-0.03274800
105	0.05714997	0.06629600
106	0.07053621	0.19233400
107	0.07053621	0.20773000
108	0.04531218	-0.09848900
109	-0.12802991	0.01394600
110	-0.03497724	0.13139000
111	-0.03497724	-0.12898000
112	-0.02094084	-0.10889100
113	0.03812466	0.35987180
114	0.35987180	-0.03760400
115	0.35987180	0.76274200
