# Supplementary material for: Evolution of tooth morphological complexity and its association with the position of tooth eruption in the jaw in non-mammalian synapsids
Source: PeerJ. 2024 Aug 12;12:e17784. doi: 10.7717/peerj.17784 (PMC11326432; doi:10.7717/peerj.17784)
Supplement: Supplemental Information 6 [file peerj-12-17784-s006.pdf]

Supplementary Information for:

Evolution of tooth morphological complexity and its association with the position of tooth eruption in the jaw in non-mammalian synapsids

Table S3: Reconstructed ancestral states of the dentition position relative to the posterior end of palatine at each node, which is numbered in Fig. S3.

Node	State at ancestor	State at node
3	0.00334064	0.00402977
4	0.00402977	-0.00225042
5	-0.00225042	-0.07972277
6	-0.07972277	-0.10223920
7	-0.10223920	-0.12824991
8	-0.12824991	-0.16832134
9	-0.16832134	-0.16385797
10	-0.16385797	-0.05261983
11	-0.05261983	-0.04533675
12	-0.04533675	-0.03805145
13	-0.03805145	0.02020480
14	0.02020480	-0.04315179
15	-0.04315179	-0.05821774
16	-0.05821774	-0.05391607
17	-0.05391607	-0.05332708
18	-0.05332708	-0.05183460
19	-0.05183460	-0.10976420
20	-0.10976420	-0.11901988
21	-0.11901988	-0.01607400
22	-0.01607400	0.02407100
23	-0.01607400	-0.05330200
24	-0.11901988	-0.16264000

25	-0.10976420	-0.08922000
26	-0.05183460	-0.00264500
27	-0.05332708	-0.05381146
28	-0.05381146	-0.03202052
29	-0.03202052	-0.06534388
30	-0.06534388	-0.05176903
31	-0.05176903	0.07326000
32	-0.05176903	-0.12918000
33	-0.06534388	-0.21259000
34	-0.03202052	-0.02832400
35	-0.05381146	-0.05862100
36	-0.05391607	-0.03598100
37	-0.05821774	-0.08427900
38	-0.04315179	-0.05703700
39	0.02020480	0.04745500
40	-0.03805145	-0.04346615
41	-0.04346615	-0.04140516
42	-0.04140516	-0.05361370
43	-0.05361370	-0.09958013
44	-0.09958013	0.05637900
45	-0.09958013	-0.06988100
46	-0.09958013	-0.20883600
47	-0.05361370	-0.01880503
48	-0.01880503	0.23874400
49	-0.01880503	-0.03152100
50	-0.04140516	-0.00762700
51	-0.04346615	-0.10053800
52	-0.04533675	-0.03394900
53	-0.05261983	-0.02977940
54	-0.02977940	-0.07508400
55	-0.02977940	0.03450100
56	-0.16385797	-0.17372197

57	-0.17372197	-0.24921602
58	-0.24921602	-0.24381421
59	-0.24381421	-0.21138183
60	-0.21138183	-0.19278262
61	-0.19278262	-0.05016600
62	-0.19278262	-0.31133500
63	-0.21138183	-0.19318400
64	-0.24381421	-0.31418400
65	-0.24921602	-0.37849897
66	-0.37849897	-0.91030000
67	-0.37849897	-0.34698800
68	-0.24921602	-0.26862026
69	-0.26862026	-0.33996600
70	-0.26862026	-0.23057600
71	-0.17372197	-0.05180300
72	-0.16832134	-0.22642222
73	-0.22642222	-0.24745848
74	-0.24745848	-0.25039337
75	-0.25039337	-0.26281458
76	-0.26281458	-0.27502078
77	-0.27502078	-0.29610743
78	-0.29610743	-0.32562350
79	-0.32562350	-0.37835740
80	-0.37835740	-0.47343147
81	-0.47343147	-0.38226900
82	-0.47343147	-0.68076300
83	-0.37835740	-0.31801100
84	-0.32562350	-0.23435700
85	-0.29610743	-0.26676147
86	-0.26676147	-0.25929400
87	-0.26676147	-0.26542500
88	-0.27502078	-0.19385500

89	-0.26281458	-0.26415700
90	-0.25039337	-0.19422900
91	-0.25039337	-0.24672200
92	-0.24745848	-0.31801000
93	-0.22642222	-0.19429417
94	-0.19429417	-0.18807800
95	-0.19429417	-0.19168400
96	-0.12824991	-0.17504170
97	-0.17504170	-0.36224273
98	-0.36224273	-0.56902900
99	-0.36224273	-0.66411700
100	-0.17504170	-0.08033900
101	-0.10223920	-0.04233981
102	-0.04233981	-0.02118284
103	-0.02118284	0.00153816
104	0.00153816	0.05305500
105	0.00153816	0.14901800
106	-0.02118284	-0.07228900
107	-0.02118284	0.03378900
108	-0.04233981	-0.23411700
109	-0.07972277	0.31820700
110	-0.00225042	0.08968500
111	-0.00225042	0.02189500
112	0.00402977	0.04309300
113	0.00334064	-0.00041320
114	-0.00041320	0.13668700
115	-0.00041320	-0.12594500
