# Supplementary material for: Evolution of tooth morphological complexity and its association with the position of tooth eruption in the jaw in non-mammalian synapsids
Source: PeerJ. 2024 Aug 12;12:e17784. doi: 10.7717/peerj.17784 (PMC11326432; doi:10.7717/peerj.17784)
Supplement: Supplemental Information 7 [file peerj-12-17784-s007.pdf]

Supplementary Information for:

Evolution of tooth morphological complexity and its association with the position of tooth eruption in the jaw in non-mammalian synapsids

Table S4: Reconstructed ancestral states of the dentition position relative to the posterior end of maxilla at each node, which is numbered in Fig. S4.

Node	State at ancestor	State at node
3	0.00795184	0.00564149
4	0.00564149	-0.00978290
5	-0.00978290	-0.09479940
6	-0.09479940	-0.10587626
7	-0.10587626	-0.11666391
8	-0.11666391	-0.20532074
9	-0.20532074	-0.20290737
10	-0.20290737	-0.00713513
11	-0.00713513	0.00673707
12	0.00673707	0.02300150
13	0.02300150	0.02561504
14	0.02561504	0.05874033
15	0.05874033	0.05325940
16	0.05325940	0.04656621
17	0.04656621	0.04421506
18	0.04421506	0.03419449
19	0.03419449	0.03020010
20	0.03020010	0.03799945
21	0.03799945	0.05863952
22	0.05863952	0.06372800
23	0.05863952	0.09558100
24	0.03799945	0.04856400

25	0.03020010	0.00630400
26	0.03419449	-0.04136000
27	0.04421506	0.09367633
28	0.09367633	0.02287802
29	0.02287802	-0.00490500
30	0.02287802	0.01508400
31	0.09367633	0.10120600
32	0.04656621	0.07354600
33	0.05325940	0.05085000
34	0.05874033	0.07361700
35	0.02561504	0.01628200
36	0.02300150	0.02803710
37	0.02803710	0.03573556
38	0.03573556	0.03724181
39	0.03724181	0.06312318
40	0.06312318	-0.01761600
41	0.06312318	0.12062500
42	0.06312318	0.05033400
43	0.03724181	0.01083141
44	0.01083141	0.06892700
45	0.01083141	0.00124100
46	0.03573556	0.08423600
47	0.02803710	0.00770800
48	0.00673707	0.02109700
49	-0.00713513	0.03096979
50	0.03096979	0.04060800
51	0.03096979	0.03847900
52	-0.20290737	-0.23115115
53	-0.23115115	-0.29744538
54	-0.29744538	-0.29397668
55	-0.29397668	-0.28617167
56	-0.28617167	-0.26855441

57	-0.26855441	-0.02667700
58	-0.26855441	-0.47591700
59	-0.28617167	-0.28518200
60	-0.29397668	-0.29745000
61	-0.29744538	-0.36914198
62	-0.36914198	-0.83890300
63	-0.36914198	-0.33361400
64	-0.29744538	-0.34252598
65	-0.34252598	-0.40639900
66	-0.34252598	-0.35709000
67	-0.23115115	-0.18402900
68	-0.20532074	-0.27261830
69	-0.27261830	-0.27989140
70	-0.27989140	-0.28406993
71	-0.28406993	-0.31859253
72	-0.31859253	-0.34525607
73	-0.34525607	-0.37156304
74	-0.37156304	-0.40507879
75	-0.40507879	-0.45761972
76	-0.45761972	-0.52150987
77	-0.52150987	-0.42198000
78	-0.52150987	-0.69538600
79	-0.45761972	-0.46513300
80	-0.40507879	-0.32997800
81	-0.37156304	-0.34716753
82	-0.34716753	-0.33527900
83	-0.34716753	-0.37136000
84	-0.34525607	-0.34851500
85	-0.31859253	-0.36766200
86	-0.28406993	-0.41312700
87	-0.28406993	0.02293300
88	-0.27989140	-0.27484900

89	-0.27261830	-0.31522618
90	-0.31522618	-0.31963600
91	-0.31522618	-0.32094800
92	-0.11666391	-0.12666880
93	-0.12666880	-0.18546379
94	-0.18546379	-0.41466400
95	-0.18546379	-0.18176400
96	-0.12666880	-0.03055900
97	-0.10587626	-0.11083401
98	-0.11083401	-0.09112015
99	-0.09112015	-0.08097081
100	-0.08097081	-0.01872200
101	-0.08097081	-0.07837200
102	-0.09112015	-0.06014300
103	-0.09112015	-0.07331300
104	-0.11083401	-0.33377600
105	-0.09479940	-0.02620800
106	-0.00978290	-0.01883800
107	-0.00978290	0.08479700
108	0.00564149	0.09000700
109	0.00795184	0.02053700
110	0.02053700	0.01206200
111	0.02053700	0.02984400
