# Supplementary material for: Evolution of tooth morphological complexity and its association with the position of tooth eruption in the jaw in non-mammalian synapsids
Source: PeerJ. 2024 Aug 12;12:e17784. doi: 10.7717/peerj.17784 (PMC11326432; doi:10.7717/peerj.17784)
Supplement: Supplemental Information 8 [file peerj-12-17784-s008.pdf]

Supplementary Information for:

Evolution of tooth morphological complexity and its association with the position of tooth eruption in the jaw in non-mammalian synapsids

Table S5: Reconstructed ancestral states of the dentition position in the cranium at each node, which is numbered in Fig. S5.

Node	State at ancestor	State at node
3	0.00843988	-0.00776594
4	-0.00776594	-0.01762766
5	-0.01762766	-0.14408299
6	-0.14408299	-0.16417589
7	-0.16417589	-0.18338817
8	-0.18338817	-0.26482391
9	-0.26482391	-0.25982822
10	-0.25982822	-0.11038409
11	-0.11038409	-0.11284098
12	-0.11284098	-0.09396514
13	-0.09396514	-0.14505199
14	-0.14505199	-0.07675403
15	-0.07675403	-0.12589158
16	-0.12589158	-0.08496551
17	-0.08496551	-0.07965003
18	-0.07965003	-0.06689234
19	-0.06689234	0.01743232
20	0.01743232	0.04524039
21	0.04524039	0.00721090
22	0.00721090	-0.00713100
23	0.00721090	0.01364100
24	0.04524039	0.11013200

25	0.01743232	-0.05547800
26	-0.06689234	-0.02206400
27	-0.07965003	-0.08292574
28	-0.08292574	0.14453111
29	0.14453111	0.00610109
30	0.00610109	0.00399867
31	0.00399867	-0.06370700
32	0.00399867	0.07684800
33	0.00610109	-0.06276300
34	0.14453111	0.16871000
35	-0.08292574	-0.13134800
36	-0.08496551	0.09669400
37	-0.12589158	-0.23392200
38	-0.07675403	-0.02450600
39	-0.14505199	-0.17268700
40	-0.09396514	-0.08084920
41	-0.08084920	-0.07242723
42	-0.07242723	-0.07498839
43	-0.07498839	-0.03679253
44	-0.03679253	-0.27916400
45	-0.03679253	-0.01897900
46	-0.03679253	0.04983000
47	-0.07498839	-0.12003576
48	-0.12003576	-0.07910500
49	-0.12003576	-0.13198000
50	-0.07242723	-0.01242900
51	-0.08084920	-0.04501400
52	-0.11284098	-0.18217000
53	-0.11038409	-0.05540199
54	-0.05540199	-0.04141100
55	-0.05540199	-0.04466500
56	-0.25982822	-0.27508145

57	-0.27508145	-0.30658202
58	-0.30658202	-0.27872172
59	-0.27872172	-0.24527215
60	-0.24527215	-0.24201000
61	-0.24201000	-0.12058100
62	-0.24201000	-0.34863600
63	-0.24527215	-0.22239700
64	-0.27872172	-0.21294800
65	-0.30658202	-0.39924962
66	-0.39924962	-0.86992400
67	-0.39924962	-0.36742300
68	-0.30658202	-0.34948497
69	-0.34948497	-0.50416600
70	-0.34948497	-0.26846600
71	-0.27508145	-0.25830100
72	-0.26482391	-0.34945049
73	-0.34945049	-0.37124569
74	-0.37124569	-0.37216886
75	-0.37216886	-0.37667157
76	-0.37667157	-0.36958793
77	-0.36958793	-0.36934598
78	-0.36934598	-0.41354356
79	-0.41354356	-0.47689668
80	-0.47689668	-0.55240964
81	-0.55240964	-0.44837900
82	-0.55240964	-0.74563400
83	-0.47689668	-0.48930100
84	-0.41354356	-0.33757500
85	-0.36934598	-0.20519639
86	-0.20519639	-0.12654900
87	-0.20519639	-0.36198100
88	-0.36958793	-0.30705600

89	-0.37667157	-0.44901300
90	-0.37216886	-0.32115500
91	-0.37216886	-0.39930500
92	-0.37124569	-0.46404400
93	-0.34945049	-0.34395963
94	-0.34395963	-0.34105600
95	-0.34395963	-0.34459900
96	-0.18338817	-0.21118472
97	-0.21118472	-0.36275433
98	-0.36275433	-0.58462700
99	-0.36275433	-0.57451700
100	-0.21118472	0.00822100
101	-0.16417589	-0.17927185
102	-0.17927185	-0.15750603
103	-0.15750603	-0.12871717
104	-0.12871717	-0.00860100
105	-0.12871717	-0.03029800
106	-0.15750603	-0.11752500
107	-0.15750603	-0.32527100
108	-0.17927185	-0.43242500
109	-0.14408299	0.05560400
110	-0.01762766	0.10339000
111	-0.01762766	0.06132800
112	-0.00776594	0.00358200
113	0.00843988	0.09671769
114	0.09671769	0.08516500
115	0.09671769	0.11831200
