# Supplementary material for: Evolution of tooth morphological complexity and its association with the position of tooth eruption in the jaw in non-mammalian synapsids
Source: PeerJ. 2024 Aug 12;12:e17784. doi: 10.7717/peerj.17784 (PMC11326432; doi:10.7717/peerj.17784)
Supplement: Supplemental Information 9 [file peerj-12-17784-s009.pdf]

Supplementary Information for:

Evolution of tooth morphological complexity and its association with the position of tooth eruption in the jaw in non-mammalian synapsids

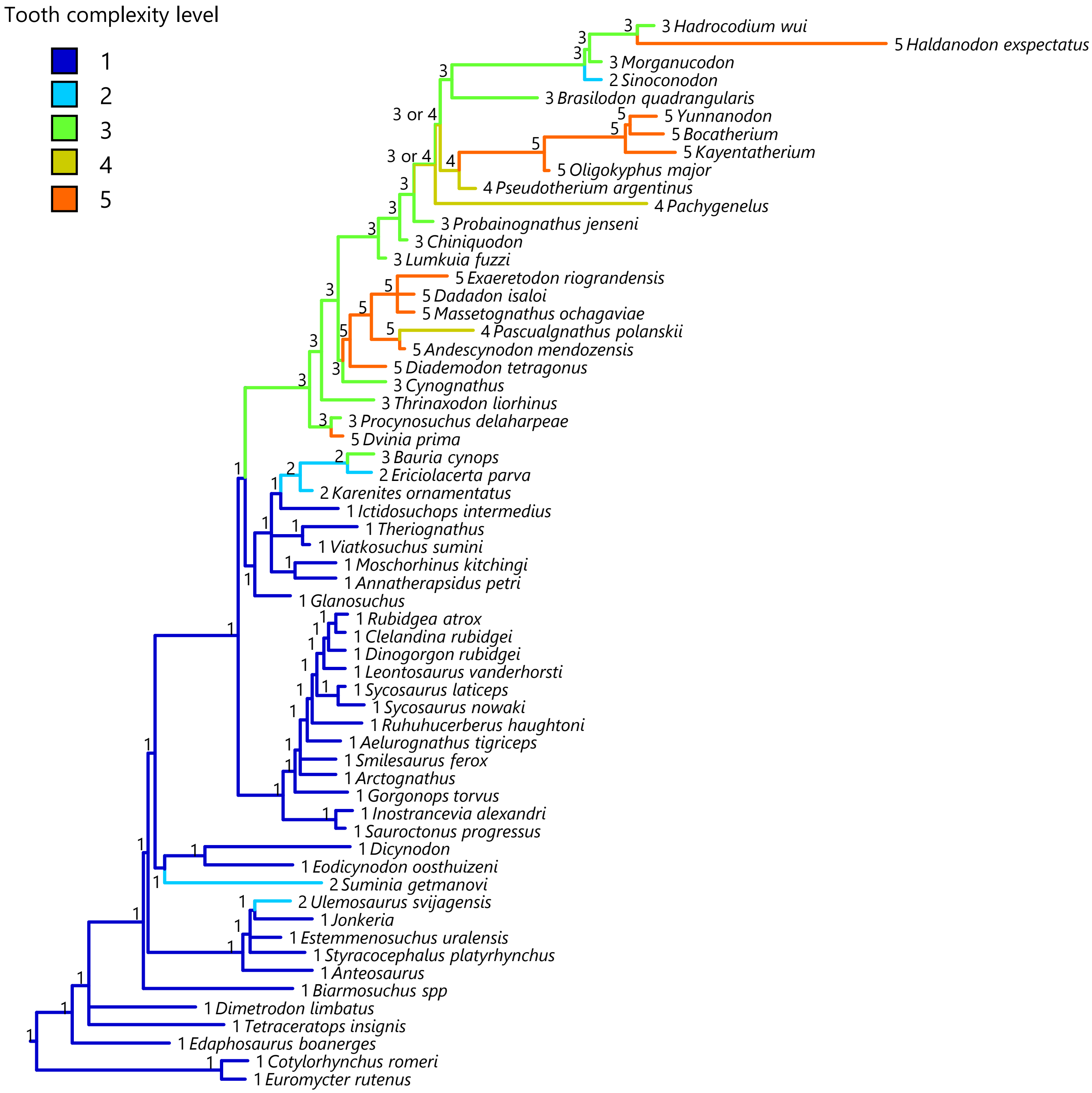

Figure S1: Evolutionary history of tooth complexity on the phylogenetic tree of non-mammalian synapsids.

The ancestral states reconstructed using parsimony methods are indicated by the colors of the branches and the values assigned to the nodes.
