# Supplementary material for: Evolution of tooth morphological complexity and its association with the position of tooth eruption in the jaw in non-mammalian synapsids
Source: PeerJ. 2024 Aug 12;12:e17784. doi: 10.7717/peerj.17784 (PMC11326432; doi:10.7717/peerj.17784)
Supplement: Supplemental Information 10 [file peerj-12-17784-s010.pdf]

Supplementary Information for:

Evolution of tooth morphological complexity and its association with the position of tooth eruption in the jaw in non-mammalian synapsids

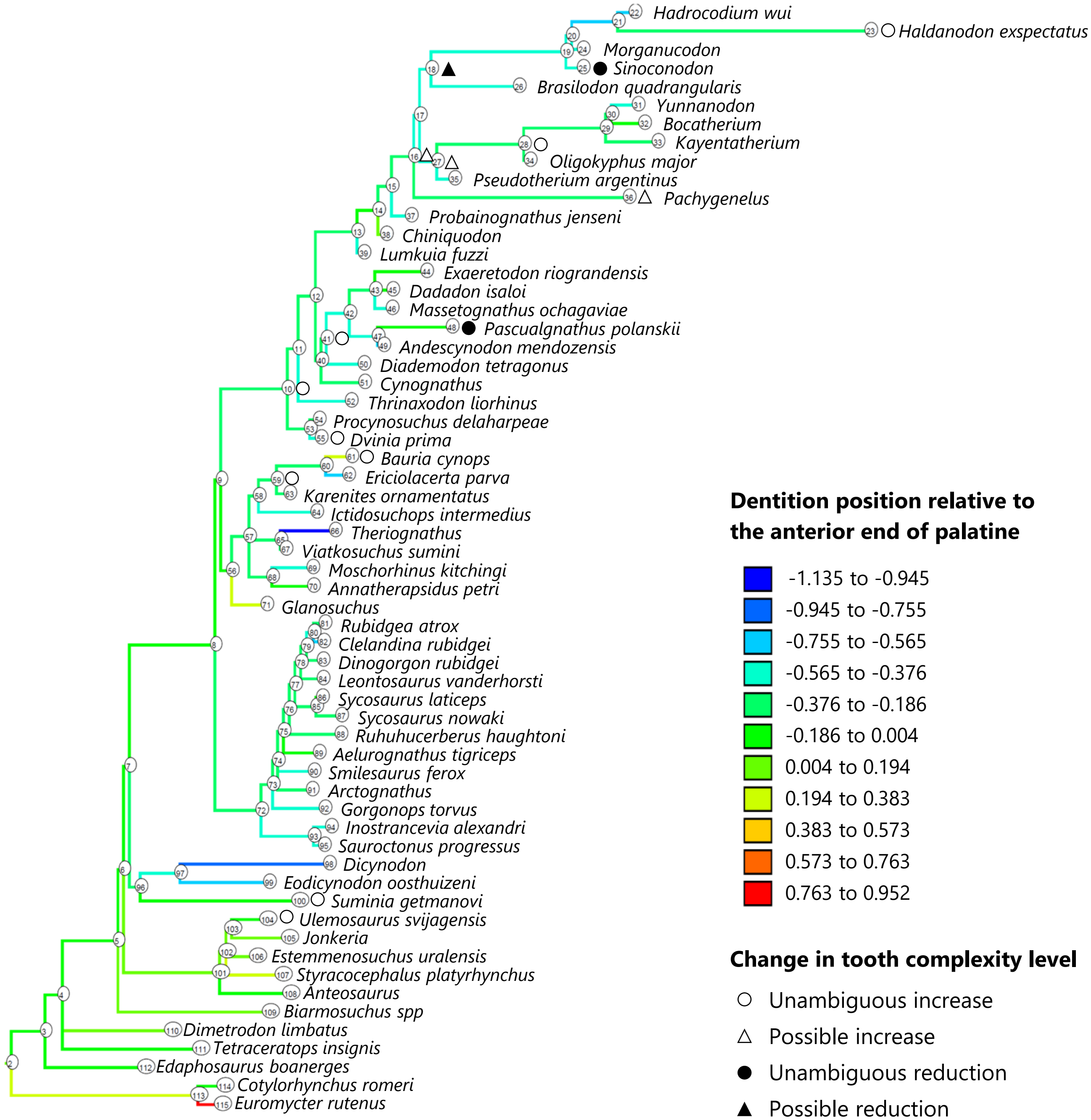

Figure S2 Evolutionary history of the dentition position relative to the anterior end of palatine on the phylogenetic tree of non-mammalian synapsids.

This position was calculated as residuals from the PGLS regression of the length to post-dentition on the length to ante-palatine. The definitions of positions and length measurements are presented in Figure 2. The statistics of the regression are presented in Table S1a. The branches are colored according to the ancestral state reconstructions using parsimony methods. The open circles represent nodes of unambiguous increased tooth complexity, the open triangles represent nodes with increased tooth complexity in one of the most likely patterns for the evolutionary history of tooth complexity, the closed circle represents a node of unambiguous reduction in tooth complexity, and the closed triangle represents a node where reduction in tooth complexity was observed in one of the most likely patterns for the evolutionary history of tooth complexity. The node numbers correspond to those of Table S2.
