# Supplementary material for: Evolution of tooth morphological complexity and its association with the position of tooth eruption in the jaw in non-mammalian synapsids
Source: PeerJ. 2024 Aug 12;12:e17784. doi: 10.7717/peerj.17784 (PMC11326432; doi:10.7717/peerj.17784)
Supplement: Supplemental Information 13 [file peerj-12-17784-s013.pdf]

Supplementary Information for:

Evolution of tooth morphological complexity and its association with the position of tooth eruption in the jaw in non-mammalian synapsids

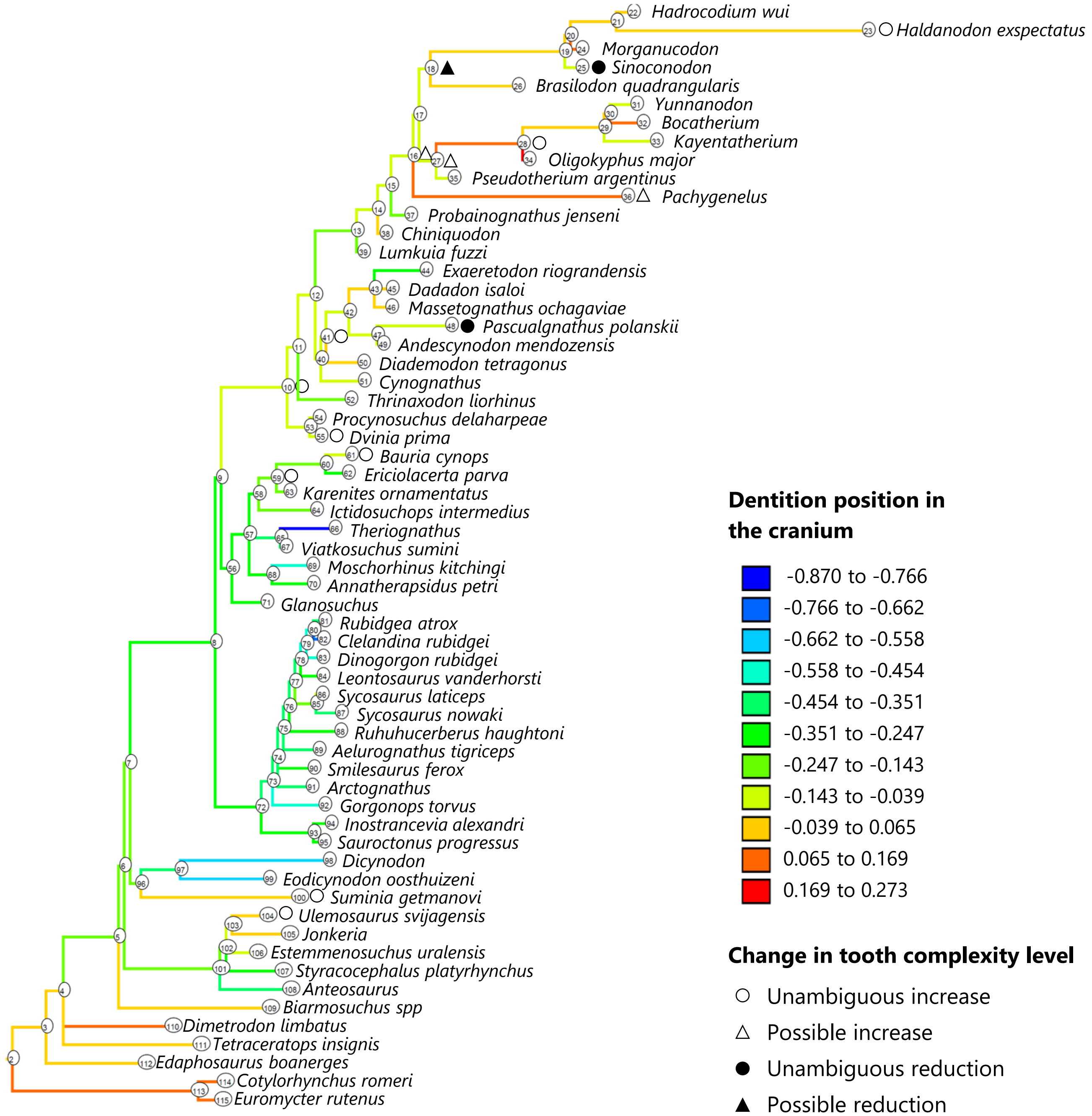

Figure S5: Evolutionary history of the dentition position in the cranium on the phylogenetic tree of non-mammalian synapsids.

This position was calculated as residuals from the PGLS regression of the length to post-dentition on the cranial length. The definitions of positions and length measurements are presented in Figure 2. The statistics of the regression are given in Table S1d. The branches are colored according to the ancestral state reconstructions using parsimony methods. The open circles, open triangles, closed circle, and closed triangle are as in Figure S2. The node numbers correspond to those of Table S5.
