# Supplementary material for: Evolution of tooth morphological complexity and its association with the position of tooth eruption in the jaw in non-mammalian synapsids
Source: PeerJ. 2024 Aug 12;12:e17784. doi: 10.7717/peerj.17784 (PMC11326432; doi:10.7717/peerj.17784)
Supplement: Supplemental Information 14 [file peerj-12-17784-s014.pdf]

Supplementary Information for:

Evolution of tooth morphological complexity and its association with the position of tooth eruption in the jaw in non-mammalian synapsids

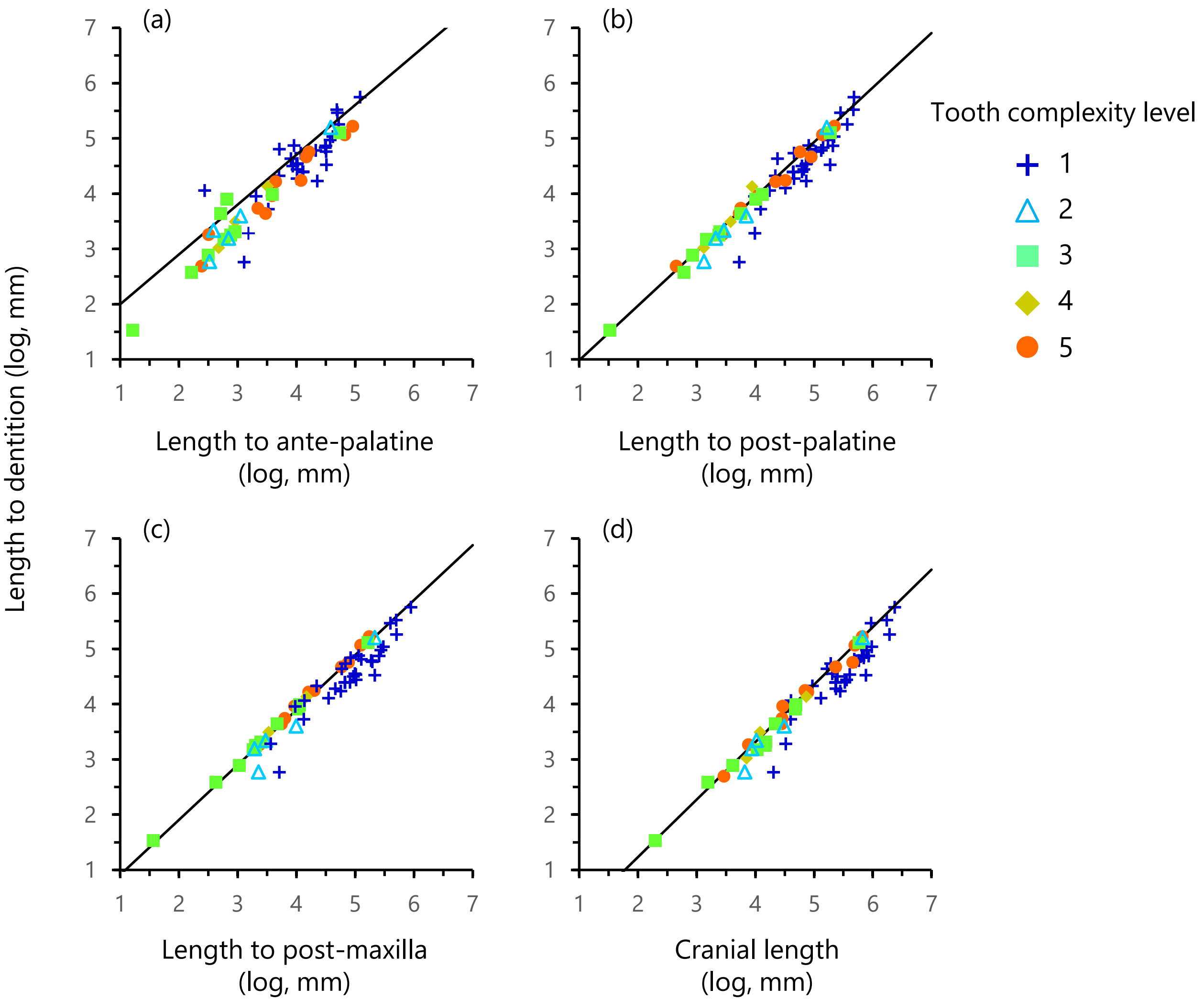

Figure S6: Length from the anterior end of the premaxilla to the point used for measuring the positioning of dentition relative to that of the reference point in the upper jaw or to the cranial length in relation to tooth complexity level in non-mammalian synapsids.

(a) The length to post-dentition relative to the length to ante-palatine, (b) the length to post-dentition relative to the length to post-palatine, (c) the length to post-dentition relative to the length to post-maxilla, and (d) the length to post-dentition relative to the cranial length. The length measurements are presented in Figure 2. The regression lines were derived from the PGLS models, from which the residuals were calculated for use in the ancestral state reconstructions of the positioning of the dentition in the upper jaw or cranium. A relatively longer length to post-dentition (a larger residual value) represents a more posterior position of dentition.
