# Supplementary material for: Evolution of tooth morphological complexity and its association with the position of tooth eruption in the jaw in non-mammalian synapsids
Source: PeerJ. 2024 Aug 12;12:e17784. doi: 10.7717/peerj.17784 (PMC11326432; doi:10.7717/peerj.17784)
Supplement: Supplemental Information 15 [file peerj-12-17784-s015.pdf]

Supplementary Information for:

Evolution of tooth morphological complexity and its association with the position of tooth eruption in the jaw in non-mammalian synapsids

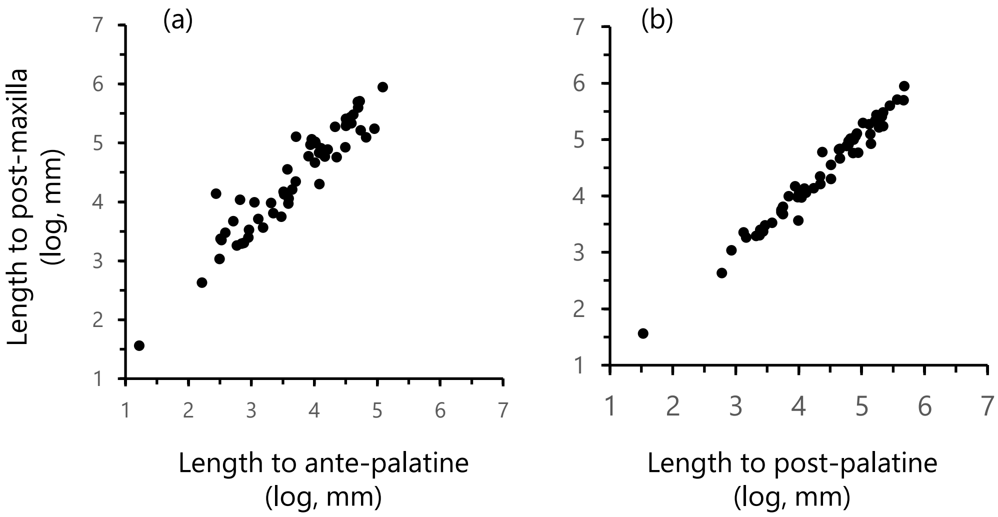

Figure S7: The length from the anterior end of the premaxilla to the posterior end of maxilla (length to post-maxilla) relative to the length from the anterior end of the premaxilla to (a) the anterior end of palatine (length to ante-palatine) or (b) the posterior end of palatine (length to post-palatine) in non-mammalian synapsids.

The length measurements are presented in Figure 2. Variance in the relative length represents variation in the positional relationships between the posterior end of maxilla and the anterior or posterior end of palatine in the upper jaw among the taxa.
